# Inter-Sectoral Collaboration in Municipal Health Centres: A Multi-Site Qualitative Study of Supporting Organizational Elements and Individual Drivers

**DOI:** 10.5334/ijic.4196

**Published:** 2019-06-21

**Authors:** Marius Brostrøm Kousgaard, Christian Elling Scheele, Karsten Vrangbæk

**Affiliations:** 1 The Research Unit for General Practice and Section of General Practice, Department of Public Health, Faculty of Health Sciences, University of Copenhagen, DK; 2 Centre for Healthy Aging, Centre for Health Economics, Department of Public Health, Faculty of Health Sciences, University of Copenhagen, DK; 3 Centre for Healthy Aging, Centre for Health Economics and Policy, Department of Public Health, Faculty of Health Sciences, and Department of Political Science, Faculty of Social Sciences, University of Copenhagen, DK

**Keywords:** collaboration, general practice, health centres, municipalities, primary care

## Abstract

**Introduction:**

Community health centres accommodating different professional groups are expected to improve inter-sectoral collaboration between primary care providers. This study aimed to identify what has been done to support inter-sectoral collaboration between municipal professionals and general practitioners in health centres, and to explore the interactions that emerge between these professionals at the operational level.

**Methods:**

The study was a multi-site qualitative study carried out in four municipal health centres in a Danish region. The study was based on documents and qualitative interviews with general practitioners, and municipal professionals and administrators in each of the health centres. A content analysis was conducted.

**Results:**

The study found that little attention had been given to the organizational prerequisites for enhanced inter-sectoral collaboration in the health centres. Even though some health centres had employed coordinators, these did not play a significant role as facilitators of collaboration partly due to a lack of political and managerial attention. At the operational level, inter-sectoral collaboration was limited to ad hoc interactions between professionals. Although these interactions could be useful, they did not evolve into more systematic forms of collaboration.

**Conclusion:**

The gap between policy visions and actual implementation efforts found in this study suggests that a more active and focused engagement from the political-administrative level is needed if the visions of increased inter-sectoral collaboration in health centres are to be realised.

## Introduction

Across the world, health care systems are looking for ways to improve coordination and collaboration across professional and administrative boundaries. This happens in response to the problems with fragmentation caused by structural specialization as well as the demographic challenges posed by aging populations with increased incidence of patients with chronic diseases that need primary sector health services from several different providers [1,2,3]. In primary care, collaboration between providers such as general practitioners (GPs) and local community professionals is important, “especially for persons in need of comprehensive services, such as fragile elderly persons, patients with chronic illnesses, patients with substance use problems and people needing to be followed up during long-term sick leaves” [4, p. 344]. However, achieving the appropriate levels of inter-sectoral and inter-professional collaboration is difficult since actors often remain embedded in separate organizational contexts with specific regulation, priorities, norms, workflows, information systems and incentive structures [5,6].

One of the arrangements which have been suggested to facilitate collaboration across administrative boundaries is community health centres that group together several primary care professionals in a specific location. These organizations have been introduced in some countries as a “primary care service delivery model with large potential for integration and collaboration” [7, p. 227]. This is also the case in Denmark where regional and local governments have established an increasing number of municipal health centres during the last decade with political and financial support from the national level. The overall goals have been to support more coherent and localized care across administrative boundaries and create a work environment that would attract more GPs in a situation where increased specialization and centralization in the hospital field has resulted in longer travel distances to health services for patients in rural parts of the country and where some areas are having problems with insufficient GP-coverage.

A municipal health centre in Denmark usually consists of one or more buildings located at the same address and accommodating municipal social services and municipal health services (provided by nurses, health workers, physiotherapists, occupational therapists) as well as a general practice clinic whose services are specified and remunerated by a general agreement with the regional health authorities. Regional policy makers have high expectations about the impact of health centres on coordination:

“Within the health centres, efforts will be made to develop health care services which provides coherence for patients, more effective patient trajectories, a concentration of multiple professional competencies and thereby a higher quality of treatment and capacity, among other things due to the expanded opportunities for cross-professional and cross-sectoral coordination, feedback and collaboration” [8, p. 20].

Thus, one of the central policy ideas promoting local health centres is that co-location will improve overall collaboration between professionals. This assumption has some support in organizational research on the significance of spatial proximity. Hence, spatial proximity has been found to increase the quantity and quality of communication between actors [9,10] and thereby also to increase mutual trust [10]. Spatial proximity can also improve the opportunity for actors to gain insight into each other’s work procedures and work conditions, and thereby increase knowledge about when and how best to contact relevant partners [11]. Furthermore, for specific inter-professional health interventions like collaborative care for anxiety and depression, co-location has been found to support implementation by improving opportunities for face-to-face communication [12].

In spite of the significant policy interest in local health centres, few studies have explored the micro-level processes of implementation and the facilitators and barriers to achieving inter-professional collaboration in these organizations. A recent case study of a single health centre in Denmark [13] found that co-location of actors did not in itself increase systematic cross-sectoral collaboration due to various barriers including a) differences in work routines and unaligned economic incentives between professionals employed by the municipality and professionals from privately owned general practice clinics b) a lack of clarity concerning the content of collaboration and c) a lack of unified management [Ibid.]. A study from a health centre in Wales [14] also reported that systemic factors (like unaligned IT-systems) limited collaboration but the study also found that local organizational initiatives – such as the establishment of a common visitation system and a multi-professional panel for care planning – worked to support inter-agency collaboration [14]. At the same time, research on inter-organisational collaboration has supplemented the more traditional focus on structural arrangements by emphasizing how individual actors – sometimes referred to as boundary spanners [15] – can facilitate collaboration across organizational boundaries through processes of networking, brokering, direction-setting and structuring of activities [16]. Some health centres in Denmark have employed coordinators who may potentially engage in boundary spanning activities to promote inter-sectoral collaboration, but little is known about their actual work and accomplishments.

More generally, studies of inter-organizational and inter-professional collaboration have shown that higher levels of collaboration contain both functional aspects (leadership support, coordination tools, information systems), normative (shared visions and goals) and relational aspects (mutual trust and knowledge of each other) [1,17,18]. Hence, developing high levels of collaboration depends on supporting organizational elements in terms of clear strategies, agreements, resources and activities as well as emerging – and positively experienced – interactions between actors at the operative level [16,17]. However, the role of these potential drivers for change in health centres and how they relate to overall policy visions have not been sufficiently investigated [14].

On this background, the objective of the present study was to explore the following questions:

1. What supporting organizational elements are applied to realize the overall visions of increased collaboration between municipal professionals and general practice in the context of health centres?
2. What types of interactions emerge in health centres between professionals from the municipality and general practice and what drives these interactions?

### The setting: Primary care in Denmark

In Denmark health care responsibilities are shared between local and regional governments. The 98 municipalities are responsible for a broad range of health and welfare services including rehabilitation and general prevention, home care, social care and care for the citizens in old age [19]. Municipalities are financed by block grants from the state and municipal income taxes. A municipal co-payment for admission to regional hospitals was introduced in 2007. The objective is to increase incentives to develop prevention and health promotion activities at the municipal level. The five regional governments are responsible for specialized medical treatment (by hospitals and other actors such as otorhinolaryngologists; midwifes; psychiatrists) as well as parts of primary care, particularly general practice.

Regions and municipalities must negotiate agreements about coordination of health care services every four years [20]. These agreements specify mutual expectations about tasks and responsibilities in regard to patient pathways. General practitioner representatives take part in the negotiations but the specification of duties for GPs is rather vague and there are no formal sanctions if individual GPs do not adhere to the agreement.

General practice serves as the primary contact point for patients before and after hospital admission. GPs own their clinics but receive almost all of their funding from the Danish regions in a combination of capitation (30%) and fee-for-service (70%). Fees and conditions are established in the general agreement between the Organization of General Practitioners and the Danish Regions every third year. Individual regions and municipalities may negotiate additional local agreements with GPs to perform specific coordination related tasks e.g. in relation to municipal nursing homes or health centres.

## Methods and materials

The study was conducted in four health centres located in different municipalities in a Danish Region. At the time of case-selection, eight health centres (in which one or more general practice clinics were co-located) existed in the Region, and we aimed to include half of them in the study. Two of the centres were so recently established that they were not considered for inclusion. Among the remaining six centres, the Region had pointed out four centres, which the Region presumed to be engaged in using co-location as a means to increase cross-sectoral collaboration. Three of these centres were successfully recruited. As one centre refused to participate, one of the last two centres was randomly selected, and this centre agreed to participate. The four health centres were established from 2013 to 2014. We recruited general practitioners, municipal professionals and administrators as respondents from the four health centres. Respondents were identified through information on the health centres’ websites, and some of the municipal professionals were identified through dialogue with administrators from the region and the health centres. The respondents consented to participate and were offered confidentiality before commencing the interviews. An overview of respondents can be found in Table 1.

**Table 1:** Interview respondents.

Health centre	From general practice	From the municipalities

Health centre A	One GP (out of one)	One nurse (out of three) Health centre coordinator
Health centre B	Two GPs (out of two)	Two consultants from prevention services Health centre coordinator Leader of nurse home care team Leader of dementia team Leader of training team
Health centre C	Two GPs (out of four)	One physiotherapist (out of two) Two health centre nurses (out of six) Executive administrator of Social Services
Health centre D	One GP (out of three) One GP secretary (out of two)	One physiotherapist (out of three) Two nurses (out of five) Executive administrator of Health and Disease Prevention Executive administrator of Elderly and Health

Semi-structured interviews (individually or in small groups) with respondents from each of the four health centres were carried out by the first and second author from December 2015 to January 2017. The main topics of the interview guide focused on the respondents’ overall view on cross-sectoral collaboration in the health centre; organizational initiatives to increase inter-sectoral collaboration; descriptions of existing interactions relations with other actors in the health centre (particularly the relations between the municipal professionals and general practice); experienced changes in inter-sectoral working relations after moving in to the health centre; experienced need for improved cross-sectoral collaboration; experienced barriers and drivers for increased collaboration at the organizational and individual level. All interviews were recorded and transcribed.

To obtain further information about visions, goals, expectations, and specific support initiatives for the health centres, we studied the following types of documents: a) project descriptions from each of the health centres, b) agendas and minutes from municipal committee meetings and c) job postings for health centre coordinators (since these could include information about the role of the coordinators as intended by the municipalities).

Based on the research questions and the topics from the interview guide we did a content analysis [21,22] of the interview data. The analyses began by reading and re-reading the material to obtain overview of and familiarity with the data. Subsequently, data from each interview was categorized and grouped together according to the topics from the interview guide. At the same time, we were open to potentially emerging themes. We compared and grouped themes from the interviews within each health centre and subsequently across health centres in order to identify similarities as well as differences between cases. In this process we also constructed sub-categories (i.e. on various types of interactions among the professionals or on the role of health coordinators as an instrument of affecting collaboration) and connected parts of the material (about supportive organizational initiatives) to higher level constructs from the literature on inter-organizational collaboration. While the analysis was guided by the pre-given themes related to the two research questions it was still rooted in and emerging from the empirical descriptions and reflections of the respondents.

According to Danish legislation [23], no ethical approval for this type of study was required since the study was not a trial and did not involve human biological material. To preserve the anonymity of the respondents we have concealed the identity of the region and the four health centres.

## Results

### Few organizational initiatives to develop and support collaboration

Overall few organizational initiatives had been taken to support collaboration in the four health centres.

Considering normative elements of support, the regional government had articulated general visions for collaboration in the health centres (see the background section). In addition to this, each of the municipalities had formulated visions and expectations for their health centres which included a focus on cross-sectoral collaboration (see Table 2). However, the region and the municipalities had not formulated more specific objectives for collaboration and neither had they described in any detail how the visions should be realized. Also, they had not initiated processes for developing shared visions and goals between the professionals in the health centres. Only one municipal administration had taken steps towards developing shared visions and goals with the GPs in the health centre, but this project had been abandoned as the municipality had decided to direct its collaborative efforts towards all GPs in the municipality since many GPs had shown an interest in this. Therefore, the visionary seminars in the municipality were not specific for the health centre and no objectives and plans were developed specifically for the health centre.

**Table 2:** Municipal visions and expectations for the health centres in relation to collaboration.

Health centre*	Visions, goals and expectations

Health centre A	Secure “knowledge sharing” and “good collaboration” between providers Utilize resources across providers “Many aspects have to be clarified when staff and tasks are coordinated and potentially placed closer to each other. This development work has begun in some areas and will be extended so that all actors can find optimal cooperative relations to the benefit of citizens and the coherency of the relevant services”
Health centre B and Health centre C	“Collaboration across professional groups will be facilitated by the physical frames and by arranging the operations of the health centre so that an organizational basis is created for collaboration across sectors and professional groups”. Collaboration between general practitioners and home care services can be extended with a focus on “the elderly, chronically ill, and mentally ill patients” The health centre will create “improved opportunities for round table discussions, and network- and theme meetings with representatives from general practice and the municipalities”. The health centre will “try out new cross-professional and cross-sectoral forms of collaboration…” “Grouping together a number of professional groups in the same health centre [will] create better conditions for collaboration in relation to the more complex patient groups which typically require much coordination and planning. Some of the existing barriers for collaborating across professional groups and sectors are expected to be undermined as the professionals will find each other within reach” “… to make local agreements with general practitioners within the health centre, which can facilitate the [forms of] collaboration that the existing conditions in the general agreement do not enable. Under these conditions it will be easier and more natural to engage in a collaboration on reaching goals for the specific patient or patient group”
Health centre D	“The goal is to co-locate regional and municipal services thereby creating a synergy effect” “Grouping together a number of professional groups in the same health centre [will] create better conditions for collaboration in relation to the more complex patient groups which typically require much coordination and planning. Some of the existing barriers for collaborating across professional groups and sectors are expected to be undermined as the professionals will find each other within reach” “… to make local agreements with general practitioners within the health centre, which can facilitate the [forms of] collaboration that the existing conditions in the general agreement do not enable. Under these conditions it will be easier and more natural to engage in a collaboration on reaching goals for the specific patient or patient group” “Collaboration between the general practitioners and home care services will be extended in relation to the elderly patients since [the health centre] will improve opportunities for conducting network- and theme meetings with representatives from general practice and the municipalities” “…develop and test more integrated and coherent [health and social] services and create a work environment that promotes collaboration […] in relation to individual patient pathways”

* Several of the statements on visions and goals were identical in the project descriptions although the health centres were located in different municipalities. This reflects the involvement of the region in drafting the project descriptions.

Considering more functional elements of support, the municipalities and local management in the health centres had made very few efforts to support the development of collaborative relations between the municipal professionals and general practice. Thus, no local agreements on cross-sectoral collaboration had been made with the resident GPs, no forum for developing such collaboration had been established and no cross-sectoral teams had been assembled. Neither had there been any attempts to develop formal tools for coordination and information exchange. Since no forums for discussing goals and/or generating specific plans for collaboration had been established this also meant that little had been done to develop the relational aspects of collaboration. However, two of the municipalities (hosting health centre A and health centre B) had dedicated resources to support collaboration by appointing local health centre coordinators who could potentially act to facilitate various aspects of collaboration.

### The role of health centre coordinators

In health centre A, the municipality had hired a part time coordinator whose tasks among other things included engaging the professionals in developing the health centre and “creating synergy between tenants” (cf. Table 3). The coordinator perceived that her role was about facilitating contacts and information between the actors and solving simple practical problems:

“I ensure that everybody greets each other; explain their everyday life to other actors; and purvey contact information… I hand out keys and explain how to install soap dispensers”. (Coordinator, health centre A)

**Table 3:** The expectations to health centre coordinators in relation to cross-sectoral collaboration.

Health centre	Expectations to coordinators as formulated in job postings

Health centre A	“Maintaining the daily operation of the buildings; promote the health centre externally; define the direction in which the health centre should develop; involve tenants in the development of the health centre; contribute to create synergy between tenants; solve ad-hoc tasks for the municipal unit for Health and Disease Prevention”
Health centre B	“…implement new accelerated [patient] workflows that are professionally sustainable” “promote communication between general practice and municipal [health professionals] in order to avoid misunderstandings that can result in unintended events…“ “analyse one-way and to-way communication between general practitioners and [municipal health] professionals [as]… basis for establishing local cross sectoral agreements concerning digital and telephonic communication; “coordinate specially themed one-day seminars for general practice and municipal employees.”

The coordinator mentioned that the GP sometimes asked her for information about new openings on the smoking cessation courses and that she was also responsible for arranging monthly ‘house meetings’. These meetings were mostly being used to discuss practical problems in the building (such as how the fire alarm functioned) rather than how to develop cross-sectoral collaboration. Thus, she did not perceive that she was responsible for initiating new forms of collaboration:

“I am not supposed to make any plans [for cross-sectoral collaboration]. I am only a catalyst for other actors’ plans concerning collaboration…” (Coordinator, health centre A)

However, no such plans had been conceived, and none of the administrators or professionals within the health centre had contacted her with ideas for increased cross-sectoral collaboration. The facilitator believed that the main drivers for cross-sectoral collaboration were co-location and the professionals seeing the same patients – rather than her own function as coordinator.

In health centre B the municipality had hired a coordinator whose main responsibility was to assist with the implementation of the municipality’s ambitious vision for cross-sectoral collaboration. The coordinator had initially sketched out several cross-sectoral projects within the health centre focusing on treatment for alcohol abuse and preventive home visits for the elderly. However, these ideas were never implemented since the coordinator soon had to go on maternal leave. By the time a temporary replacement was hired, the municipality had decided to focus on other projects involving all the general practices in the municipality. Hence, no cross-sectoral collaborative initiatives were set up specifically for the health centre.

In health centre C and health centre D, no local coordinators were appointed when the centres were established, partly for resource reasons and partly because of expectations that co-location would be enough for achieving cross-sectoral collaboration:

“Our strategy was to provide the physical location – and then [collaboration] would have to grow bottom-up.” (Head of Elderly and Health, health centre D)

In health centre C, the executive administrator had arranged a workshop attended by various professionals within the centre (including GPs, municipal health professionals, and private health care professionals) when he had learned that the centre had not been able to generate systematic forms of cross-sectoral collaboration. Based on feedback from the workshop, he subsequently set out to develop a shared website; an intranet; a phone list; information concerning a defibrillator that non-professionals can use and clearer information concerning health centre services to citizens. In order to develop more substantial cross-sectoral collaboration, the executive administrators of health centres C and D believed that it was necessary to appoint a coordinator/facilitator with more dedicated time. Still, they had doubts about how effective a coordinator would be without a formal cross-sectoral mandate issued at the policy level. (At the time of the study, health centre C had just hired a part time coordinator, but she had not yet initiated any activities).

Overall, we found no indications that municipal politicians or higher-level administrators directly requested the health centre administrators or coordinators to engage in developing intensified cooperative relations within the health centre. Furthermore, due to their vague mandate the coordinators were hesitant to engage in coordination efforts across administrative boundaries. In practice, it was unclear which types of cross-sectoral collaboration activities they should engage in, and they were reluctant to engage in activities that would issue demands on the resources of the other actors in the health centres.

### Inter-sectoral professional interactions in the health centres

Most of the professional work in the health centres was intra-sectoral but some inter-sectoral interactions between municipal professionals and general practice were identified. Thus, many municipal professionals viewed the general practitioners as a knowledge resource which could help them in their management of specific citizens/patients. Therefore, the most common forms of inter-sectoral interactions were ad hoc interactions where the municipal professionals contacted the GPs for advice:

“…I asked the GP for advice because I was in doubt as to whether the patient had an infection and whether we should commence an antibiotic treatment, which he has to prescribe. I also had a patient with a loose nail that I needed him to look at”. (Municipal nurse, health centre A)

“…if the GP has given me instructions that I do not understand, or the instructions do not match with what the citizens says; or if [the citizen] feels pain that should not be present then I contact the GPs…. And frequently I can get immediate access to the GP”. (Physiotherapist, health centre D)

These interactions were usually face-to-face (and sometimes by phone) as the professionals took advantage of the spatial proximity offered by the health centre and the interactions could also take the form of mutual information sharing and discussion of patient cases:

Nurse (health centre D): “I think it has become much easier just to go to them [the GPs] if we have something, we would like to discuss face to face.”

I: “What could that be, for example?”

Nurse: “If we have a citizen and we have problems with the medication that has been prescribed, what we think and what they think and discuss it. [Also] the general condition of the citizen […]”.

While it was typically the municipal professionals who initiated the advisory type of interactions, a few of the GPs made frequent use of the opportunities for face-to-face interactions about individual patients if they perceived that this benefitted their work and their patients:

“I experience many advantages of being in a house with home nursing services and job services and I have taken many trips on the stairs and solved problems ad hoc, grabbed the paper and rushed down below [where] the job services, social services and home nursing services are located. Actually, if I have a specific problem, I sometimes tell the patient ‘hold on for five minutes and then I will be back’, and then I run down stairs.” (GP1, health centre B)

Sometimes, this GP also engaged in more formal round table discussions with social services as a way of bypassing what was perceived as bureaucratic paper work and of making decisions together with the patients and the relevant municipal actors:

“I had a round table discussion today with a social worker, and a patient who had been in an accident… I see that as a time-effective way of working rather than writing a lot of documents and sending requests for new documents round in the system and so.” (GP1, health centre B)

Similarly, another GP related that easy access to the municipal professionals resulted in more effective problem solving:

”…the professionals that I collaborate with – home nurses, physiotherapist, dietician and social workers are here at the centre […] there is a fine collaboration; well, it is only about my own patients that I can talk to the nurses but the chain of command is very short and we can usually fix it the same day or the same hour if there are any problems…” (GP, health centre A)

Discussing specific patients gradually made the professionals more acquainted with each other’s ways of working and some of the GPs found it easier (both for themselves and their patients) to refer to the municipal services in the centre which they knew and which were ‘right next door’:

“If I have a patient with a wound, the wound ambulatory is just down below where the nurses are. They can take over the patient, so I do not have to do it […] If I have a nutritional problem related to diabetes then I have a dietician which I can contact quickly and then she can take the patient in. And it is an advantage for the patients that they only have to attend one treatment setting. So, we all benefit.” (GP, health centre A)

The municipal professionals also contacted the GPs in acute situations in which the proximity and consequently rapid response of the GPs was much appreciated:

“We have had some extreme cases where it was really useful to have a doctor in the centre. For instance, we had a citizen who suddenly fell ill during training and we were able to get a doctor very fast rather than having to wait for someone coming from outside. There have been a couple of cases where we had to call the emergency line, and, in those situations, it was fantastic to have the doctor here because the doctor is just really quick at diagnosing and describing the patient’s condition to the emergency service”. (Leader of home nursing, health centre B)

“[Having a GP in the health centre] really means quite a lot. It is often less complicated for me to get to the GP if there is an emergency. Sometimes GPs reroute their phone line to colleagues in different parts of the town during midday – but I know that they are still there, and I can access them if I have an emergency. It is much easier communicating face to face than over the phone.” (Nurse, health centre C)

There was considerable variation among the professionals in their tendency to initiate the types of cross-sectoral interactions described above. Such differences were related to the professionals’ attitudes to engaging in face-to-face interactions which were affected by individual traits, past experiences with collaboration, perceptions of time-effectiveness, or considerations about the appropriateness of verbal vs. written (electronic) communication, and – in one case – issues of conflicting professional interests. As illustrated above some professionals enjoyed the relational aspects and advantages of face-to-face interactions. However, some of the municipal professionals were more sceptical as they preferred communication to be in writing because this increased the likelihood of the GPs getting in contact with the right person, or because the formality of such correspondence was more likely to ensure that the ideal of equality in services was not compromised:

“There should be no differences in the way we work with the GPs [in the area]. So just because you have a GP in this centre you should not be better off than if you have a GP on street [X] or street [Y]. It has to be equal for everybody”. (Leader of physiotherapy, health centre B)

Furthermore, the same respondent mentioned that one of the GPs was sometimes too demanding when he wanted municipal approval for specific services for his patients:

“…we feel that the doctor thinks that it is okay for him to exert pressure to secure a place for his citizen in one of our residence services [for rehabilitation, training, or home care]. And then it suddenly becomes too… then it is not a dialogue but more like a command.” (Leader of physiotherapy, health centre B)

Here, following standard procedures of written communication was seen by some municipal actors as a way to avoid face-to-face interactions in which the professionals might not agree about what was to be done and where the GP would have the advantage in terms of professional authority (although there was no formal hierarchy in relations between the GPs and the municipal professionals since they belonged to different organizations).

None of the professionals had made attempts to extend cross-sectoral co-operations beyond the case-by-case interactions described above. Some of the GPs expressed regret about this but at the same they were reluctant about participating in regular meetings as part of a broader developmental agenda since this would take time away from their patients and for some potentially decrease job satisfaction:

“It has not become a coordinated effort – I believe much more of that could be done… [But] I think there is more satisfaction [in doing something for the patients] […] It is about seeing as many patients as possible – not because of the economic aspect – it is simply because the patients stand [in line] knocking on your door […] and I find it stressing to tell them that they can come back in a week or three. It is nice to say, ‘come back tomorrow or whenever you have time or right now’”. (GP, health centre D)

For other GPs, the lack of time combined with a lack of will (on part of the municipality and region) to pay for time spent on developing and implementing cross-sectoral collaboration impeded their engagement.

Summing up, physical proximity in the health centres was regarded as beneficial by many respondents. However, the increased collaboration had mostly been intra-sectoral among the different municipal health and care staff. To the extent that cross-sectoral collaboration was mentioned it was mostly between GPs and municipal nurses, and focused on information exchange around specific patients, or on specific events such as emergencies. Here, easy access was mentioned as a key benefit. There were no signs of more comprehensive joint planning of activities or systematic sharing of information.

## Discussion

This study set out to explore organizational strategies to support the visions of increased inter-sectoral collaboration between municipal health services and general practice in four health centres, and to identify what kinds of professional interactions emerged in the centres. The study found that very few governance initiatives had been taken to support inter-sectoral collaboration in the health centres: No specific objectives were formulated, no formal development forums were set up, no formal teams were established, no formal agreements were made, and no particular incentives were provided. Apparently, the political and administrative actors at the regional and municipal had not been very engaged in creating effective organizational conditions for cross-sectoral collaboration. This finding contrasts with the relatively high ambitions for cross-sectoral collaboration expressed in the official discourse on the health centres. This gap between rhetoric and specific action at the policy level seems to have two related explanations: First, the regional and municipal policy actors simply had overly optimistic expectations about the ability of the health centres to drive cross-sectoral collaboration forward via the mechanism of co-location. Second, developing more cross-sectoral collaboration inside the health centres was in practice deemed less important (thereby receiving less attention and resources) than other policy goals such as a) securing the recruitment of GPs to the health centres in the first place (an important and easily evaluable criterion for receiving national funding); b) planning and carrying out the installation of the various municipal, private and regional providers in the health centres; or c) achieving improved collaborative relations with all GP-clinics in the municipality rather than only with those in the health centre (as in health centre B). This second explanation conforms to institutionalist theories which emphasize the often symbolic nature of official rhetoric and decisions in organizations [24,25]. On the one hand, organizations have an interest in appearing modern and innovative to their stakeholders by adopting new ideas and technologies. On the other hand, actual implementation is often difficult and costly (particularly in political or professionally dominated organizations with competing goals and/or high levels of task complexity), and this tension may result in decoupling between rhetoric and operations. The question is whether such decoupling is maintained over time or steps will be taken to attain more alignment between policies and practices [26].

The only potential organizational support element for increasing inter-sectoral collaboration that we identified in this study was the employment of health centre coordinators in two of the municipalities. But while the coordinators to some extend facilitated information among the professionals and solved some technical problems, they did not become effective facilitators for the development of new collaborative relations between the municipal services and general practice. This was partly due to the weak formal mandate of the coordinators which was a consequence of the limited engagement of the political-administrative actors in charge of the health centres. The implications of this were that neither the coordinators themselves nor the stakeholders had a clear picture of the competencies and tasks related to their role. Previous literature on boundary spanners in health care also suggests that it is difficult for individual actors without specified objectives and a strong formal mandate to significantly impact systematic collaboration across administrative borders [27].

Concerning cross-sectoral collaboration at the operational level in the health centres, the professionals reported several examples where they had used the physical proximity offered by the health centres to engage in ad hoc, face-to-face interactions in order to deal with specific patient cases. In the terminology of Boon et al. [28] these interactions were usually consultative in nature, but sometimes they also involved mutual exchange of information and discussions of treatment options. A recent study among nurses in community and acute settings also found that the professionals appreciated the relational advantages of face-to-face communication offered by co-location [29]. However, in our cases cross-sectoral interactions were mostly sporadic, and most professional work was carried out separately in the two sectors within the health centres. There were no examples of the development of more systematic and formalized cross-sectoral collaborative interactions that were substantially different from current practices outside of the health centres. Both the municipal professionals and the professionals from general practice sustained their traditional focus on the clinical tasks of diagnosing and treating individual patients and did not take on additional roles in developing formal collaboration across sectors. In the absence of active organizational strategies, two factors were decisive for the initiation of cross-sectoral interactions in the health centres: a) the personal attitudes and preferences of the professionals and b) individual professionals’ perceptions that the knowledge and competencies of other actors in the health centre were important for their own task completion, i.e. resource dependency as a driver [30]. Similar factors affecting the dynamics of inter-professional interactions have been found in a study by MacNaughton et al. [31] who also emphasize the importance of individual attributes and perceived relevance of other actors’ professional knowledge. While that study focused on formally established teams, our study concerned interactions that emerge when professionals from different sectors are co-located without a strong governance frame to direct, authorize, and incentivize cross-sectoral collaboration in conjunction with the potentials of co-location.

Overall there were marked variations in the individual professionals’ inclination to engage in collaborative relations. Similarly, a recent study from Norway reported “striking individual differences” in GPs attitudes and behaviours concerning collaboration with municipal professionals [4, p. 348]. The authors elaborate that GPs have to prioritize their time between a large number of patients with relatively simple problems and a lesser number of patients who needs a coordinated effort, and that different professional identities among the GPs seem to be of great significance to this prioritization [4]. Variations among the municipal professionals in our study were partly related to the fact that some had concerns over the legality of making special collaborative arrangements within the health centre so that citizens connected to the centre would receive better care than citizens not attending the centre. To our knowledge this type of barrier has not previously been reported, but it is an important issue that should be addressed at the administrative-political level and clearly communicated to staff.

The gap between policy ambitions and organizational implementation found in this study indicates that barriers for increased collaboration are stronger than the impetus provided by co-location within health centres – at least in the short run. Therefore, if higher levels inter-sectoral collaboration is a serious political ambition, more attention should be paid to developing strategies containing specific tools and objectives (rather than vague visions). Strategies should be developed by representatives from both two sectors and prioritize collaborative arrangements with the most potential for benefitting patients in primary care. Incentives for participation in cross-sectoral collaboration should be provided through local agreements. Although health centre coordinators may be a way to facilitate collaboration, it is important to consider their mandate and to clarify role expectations. Finally, such strategies should include an assessment of whether the goals of collaboration are achieved, what the costs are, and how to follow up with appropriate organizational actions.

### Limitations

This study provides a picture of the current state of inter-sectoral collaboration in four health centres which had been established two to three years prior to data collection. At the time of the study, the level of formal, systematic inter-sectoral collaboration – beyond what was already happening outside the health centres – was low. It is possible that collaboration in the health centres may evolve to higher levels over time e.g. due to increased political pressure and support from the municipal and/or regional level. A recent news report [32] from a health centre in another region suggests that inter-sectoral collaboration may improve further than what was found in our cases. Studying more examples of apparently successful cases could produce more knowledge about the significant drivers of increased collaboration in these settings. Methodologically, the study relied on documents and interviews. A more ethnographic approach (observing specific interactions in the health centres over time) might have yielded a richer analysis of the emergence, content and dynamics of inter-sectoral collaborations.

In regards to the overall policy ambitions of integrated care, this study only looked at collaboration within parts of primary care in the specific context of health centres. However, for many patients, integrated care also involves providers from secondary care. In this perspective, the difficulties found in the present study underline the extent and ‘wickedness’ [33] of the overall challenge for health care systems to achieve integrated care across professions, organizations and sectors.

## Conclusion

This study addressed two questions. The first question concerned supportive organizational elements in health centres to realize the visions of increased collaboration between municipal professionals and general practice. The study found that very few supportive organizational elements had been designed and that the engagement of the political-administrative actors in advancing cross-sectoral collaboration in the health centres had been surprisingly low. We suggest that this weak coupling between policy rhetoric and supportive organizational actions is a result of competing political-administrative priorities related to the health centres. The second question concerned the emergence of interactions between professionals from the municipality and general practice and the driving forces behind such interactions. Our analysis showed that most of the interaction was intra-sectoral, although some examples of fruitful interaction across sectors at the operational level were identified. These interactions were mainly ad hoc and consultative and driven by resource dependencies (especially with knowledge and competencies as important resources), and there was little integration of planning and delivery of health care activities. This leads us to conclude that a much more directed political effort – involving a clear and detailed strategy supported by local agreements – is necessary if health centres are to become generators for more advanced forms of inter-sectoral collaboration.

## References

[1] Valentijn, PP, Schepman, SM, Opheij, W and Bruijnzeels, MA. Understanding integrated care: a comprehensive conceptual framework based on the integrative functions of primary care. International Journal of Integrated Care, 2013; 13(1). DOI: 10.5334/ijic.886 PMC3653278 23687482

[2] Bodenheimer, T. Coordinating Care – A Perilous Journey through the Health Care System. The New England Journal of Medicine, 2008; 358(10): 1064–1071. DOI: 10.1056/NEJMhpr0706165 18322289

[3] Colmorten, E, Clausen, T and Bengtsson, S. Providing integrated health and social care for older persons in Denmark In: Leichsenring K, Alaszewski AM, (eds.). Providing integrated health and social care for older persons – A European review of issues at stake, 2004; p. 139–80 Vienna: European Centre and Aldershot: Ashgate Publishing.

[4] Steihaug, S, Paulsen, B and Melby, L. Norwegian general practitioners’ collaboration with municipal care providers – a qualitative study of structural conditions. Scandinavian Journal of Primary Health Care, 2017; 35(4): 344–351. DOI: 10.1080/02813432.2017.1397264 29116877 PMC5730032

[5] Supper, I, Catala, O, Lustman, M, Chemla, C, Bourgueil, Y and Letrilliart, L. Interprofessional collaboration in primary health care: a review of facilitators and barriers perceived by involved actors. Journal of Public Health, 2014; 37: 716–727. DOI: 10.1093/pubmed/fdu102 25525194

[6] Auschra, C. Barriers to the Integration of Care in Inter-Organisational Settings: A Literature Review. International Journal of Integrated Care, 2018; 18(1): 5 DOI: 10.5334/ijic.3068 PMC5887071 29632455

[7] Suter, E, Hyman, M and Oelke, N. Measuring key integration outcomes: A case study of a large urban health center. Health Care Management Review, 2007; 32(3): 226–235. DOI: 10.1097/01.HMR.0000281624.43611.dd 17666993

[8] Praksisplanudvalget i Region Syddanmark: Praksisplan for almen praksis 2015–18 [Practice Plan for General Practice 2015–18] Region Syddanmark 2015. [in Danish].

[9] Knoben, J and Oerlemans, LA. Proximity and inter-organizational collaboration: A literature review. International Journal of Management Reviews, 2006; 8(2): 71–89. DOI: 10.1111/j.1468-2370.2006.00121.x

[10] Gössling, T. Proximity, trust and morality in networks. European Planning Studies, 2004; 12(5): 675–689. DOI: 10.1080/0965431042000220011

[11] Monge, PR, Rothman, LW, Eisenberg, EM, Miller, KI and Kirste, KK. The dynamics of organizational proximity. Management Science, 1985; 31(9): 1129–1141. DOI: 10.1287/mnsc.31.9.1129

[12] Overbeck, G and Davidsen, AS and Kousgaard MB. Enablers and barriers to implementing collaborative care for anxiety and depression: a systematic qualitative review. Implementation Science, 2016; 11: 165 DOI: 10.1186/s13012-016-0519-y 28031028 PMC5192575

[13] Scheele, C and Vrangbæk, K. Co-location as a Driver for Cross-Sectoral Collaboration with General Practitioners as Coordinators: The Case of a Danish Municipal Health Centre. International Journal of Integrated Care, 2016; 16(4). DOI: 10.5334/ijic.2471 PMC5354220 28316555

[14] Kaehne, A and Catherall, C. Co-located health and social care services in Wales: What are the benefits to professionals? International Journal of Health Care Management, 2012; 5(3): 164–172. DOI: 10.1179/2047971912Y.0000000014

[15] Williams, P. Collaboration in public policy and practice: Perspectives on boundary spanners Bristol: The Policy Press; 2012 DOI: 10.2307/j.ctt1t89g31

[16] Delaney, FG. Muddling through the middle ground: theoretical concerns in intersectoral collaboration and health promotion. Health Promotion International, 1994; 9(3): 217–225. DOI: 10.1093/heapro/9.3.217

[17] D’Amour, D, Goulet, L, Labadie, JF, Martín-Rodriguez, LS and Pineault, R. A model and typology of collaboration between professionals in healthcare organizations. BMC Health Services Research, 2008; 8: 188 DOI: 10.1186/1472-6963-8-188 18803881 PMC2563002

[18] Gittell, JH, Godfrey, M and Thistlethwaite, J. Interprofessional collaborative practice and relational coordination: improving healthcare through relationships. Journal of Interprofessional Care, 2013; 27(3): 210–213. DOI: 10.3109/13561820.2012.730564 23082769

[19] Olejaz, M, Juul Nielsen, A, Rudkjøbing, A, Okkels Birk, H, Krasnik, A and Hernández-Quevedo, C. Denmark health system review. Health Systems in Transition, 2011; 14(2): i–xxii, 1–192. 22575801

[20] Rudkjøbing, A, Strandberg-Larsen, M, Vrangbæk, K, Andersen, JS and Krasnik, A. Health care agreements as a tool for coordinating health and social services. International Journal of Integrated Care, 2014; 14(4). DOI: 10.5334/ijic.1452 PMC4276032 25550691

[21] Hsieh, HF and Shannon, SE. Three Approaches to Qualitative Content Analysis. Qualitative Health Research, 2005; 15(9): 1277–1288. DOI: 10.1177/1049732305276687 16204405

[22] Elo, S and Kyngäs, H. The qualitative content analysis process. Journal of Advanced Nursing, 2008; 62: 107–115. DOI: 10.1111/j.1365-2648.2007.04569.x 18352969

[23] Act on Research Ethics Review of Health Research Projects. Available from: http://www.nvk.dk/english/act-on-research [cited May 29, 2018].

[24] Brunsson, N. The Organization of Hypocrisy Talk, Decisions and Actions in Organizations, 2nd ed. Oslo: Universitetsforlaget AS; 2006.

[25] Meyer, JW and Rowan, B. Institutionalized Organizations: Formal Structure as Myth and Ceremony. American Journal of Sociology, 1977; 83(2): 340–363. DOI: 10.1086/226550

[26] Bromley, P and Powell, WW. From Smoke and Mirrors to Walking the Talk: Decoupling in the Contemporary World. Academy of Management Annals, 2012; 6(1): 483–530. DOI: 10.1080/19416520.2012.684462

[27] Kousgaard, MB, Joensen, ASK and Thorsen, T. The challenges of boundary spanners in supporting inter-organizational collaboration in primary care – a qualitative study of general practitioners in a new role. BMC Family Practice, 2015; 16: 17 DOI: 10.1186/s12875-015-0231-z 25887910 PMC4355472

[28] Boon, H, Verhoef, M, O’Hara, D and Findlay, B. From parallel practice to integrative health care: a conceptual framework. BMC Health Services Research, 2004; 4(1): 15 DOI: 10.1186/1472-6963-4-15 15230977 PMC459233

[29] King, N, Bravington, A, Brooks, J, Melvin, J and Wilde, D. ‘Go Make Your Face Known’: Collaborative Working through the Lens of Personal Relationships. International Journal of Integrated Care, 2017; 17(4): 3 DOI: 10.5334/ijic.2574 PMC5624119 28970761

[30] McDonald, J, Jayasuriya, R and Harris, MF. The influence of power dynamics and trust on multidisciplinary collaboration: a qualitative case study of type 2 diabetes mellitus. BMC Health Services Research, 2012; 12: 63 DOI: 10.1186/1472-6963-12-63 22413897 PMC3376040

[31] MacNaughton, K, Chreim, S and Bourgeault, IL. Role construction and boundaries in interprofessional primary health care teams: a qualitative study. BMC Health Services Research, 2013; 13: 486 DOI: 10.1186/1472-6963-13-486 24267663 PMC4222600

[32] Pinborg, K. Nær sammenhæng i sundhedshus skaber mening for borgere og sundhedspersoner. [Proximity in health center makes sense for citizens and professionals]. Dagens Medicin 24.08.2017. Available from: https://kommunalsundhed.dk/naer-sammenhaeng-sundhedshus-skaber-mening-patienter-fagpersoner/ [cited March 19, 2018]. [in Danish].

[33] Shaw, SE and Rosen, R. Fragmentation: a wicked problem with an integrated solution? Journal of Health Services Research & Policy, 2013; 18(1): 61–4. DOI: 10.1258/jhsrp.2012.012002 23393045

